# Supplementary material for: Gβγ engages PLCβ3 at multiple sites to reorient and facilitate its activation
Source: bioRxiv. 2026 Jan 14:2026.01.14.699417. Preprint. [Version 1] doi: 10.64898/2026.01.14.699417 (PMC12871268; doi:10.64898/2026.01.14.699417)
Supplement: 1 [file NIHPP2026.01.14.699417V1-supplement-1.pdf]

bioRxiv preprint doi: https://doi.org/10.64898/2026.01.14.699417; this version posted January 16, 2026. The copyright holder for this preprint (which was not certified by peer review) is the author/funder, who has granted bioRxiv a license to display the preprint in perpetuity. It is made available under aCC-BY 4.0 International license.

Supplemental Information

Appendix: Materials and Methods

Supplementary References

48. R. G. de Rubio *et al.*, Phosphatidylinositol 4-phosphate is a major source of GPCR-stimulated phosphoinositide production. *Science Signaling* **11** (2018).
49. T. L. Davis, T. M. Bonacci, S. R. Sprang, A. V. Smrcka, Structural and molecular characterization of a preferred protein interaction surface on G protein βγ subunits. *Biochemistry* **44**, 10593-10604 (2005).
50. T. Kozasa, "Purification of recombinant G protein α and βγ subunits from Sf9 cells" in G Proteins : Techniques of Analysis, D. R. Manning, Ed. (CRC Press LLC, 1999), pp. 23-37.
51. M. Ghosh, A. V. Smrcka, Assay for G protein-dependent activation of phospholipase C β using purified protein components. *Methods Mol Biol* **237**, 67-75 (2004).
52. M. Ghosh, H. Wang, G. G. Kelley, A. V. Smrcka, Purification of phospholipase C β and phospholipase C ε from Sf9 cells. *Methods Mol Biol* **237**, 55-64 (2004).
53. A. Punjani, J. L. Rubinstein, D. J. Fleet, M. A. Brubaker, cryoSPARC: algorithms for rapid unsupervised cryo-EM structure determination. *Nature Methods* **14**, 290-296 (2017).
54. L. G. Trabuco, E. Villa, E. Schreiner, C. B. Harrison, K. Schulten, Molecular dynamics flexible fitting: A practical guide to combine cryo-electron microscopy and X-ray crystallography. *Methods* **49**, 174-180 (2009).
55. A. Casañal, B. Lohkamp, P. Emsley, Current developments in Coot for macromolecular model building of Electron Cryo-microscopy and Crystallographic Data. *Protein Science* **29**, 1055-1064 (2020).
56. P. D. Adams *et al.*, PHENIX: a comprehensive Python-based system for macromolecular structure solution. *Acta Crystallogr D Biol Crystallogr* **66**, 213-221 (2010).
57. D. Liebschner *et al.*, Macromolecular structure determination using X-rays, neutrons and electrons: recent developments in Phenix. *Acta Crystallographica Section D Structural Biology* **75**, 861-877 (2019).
58. W. Jang, K. Senarath, G. Feinberg, S. Lu, N. A. Lambert, Visualization of endogenous G proteins on endosomes and other organelles. *eLife* **13** (2024).
59. J. C. Madukwe, E. E. Garland-Kuntz, A. M. Lyon, A. V. Smrcka, G protein betagamma subunits directly interact with and activate phospholipase Cepsilon. *J Biol Chem* 10.1074/jbc.RA118.002354 (2018).
60. T. Kozasa, A. Gilman, Purification of recombinant G proteins from Sf9 cells by hexahistidine tagging of associated subunits. Characterization of a_{12} and inhibition of adenylyl cyclase by a_z. *J Biol Chem* **270**, 1734-1741 (1995).
61. S. Q. Zheng *et al.*, MotionCor2: anisotropic correction of beam-induced motion for improved cryo-electron microscopy. *Nature Methods* **14**, 331-332 (2017).
62. A. Rohou, N. Grigorieff, CTFFIND4: Fast and accurate defocus estimation from electron micrographs. *Journal of Structural Biology* **192**, 216-221 (2015).
63. M. A. Wall *et al.*, The structure of the G protein heterotrimer $G_i\alpha_1\beta_1\gamma_2$. *Cell* **83**, 1047-1058 (1995).
64. E. F. Pettersen *et al.*, UCSF Chimera—A visualization system for exploratory research and analysis. *J Comput Chem* **25**, 1605-1612 (2004).

bioRxiv preprint doi: https://doi.org/10.64898/2026.01.14.699417; this version posted January 16, 2026. The copyright holder for this preprint (which was not certified by peer review) is the author/funder, who has granted bioRxiv a license to display the preprint in perpetuity. It is made available under aCC-BY 4.0 International license.

65. G. Terashi, X. Wang, D. Kihara, Protein model refinement for cryo-EM maps using AlphaFold2 and the DAQ score. *Acta Crystallographica Section D Structural Biology* **79**, 10-21 (2023).
66. G. Terashi, X. Wang, S. R. Maddhuri Venkata Subramaniya, J. J. G. Tesmer, D. Kihara, Residue-wise local quality estimation for protein models from cryo-EM maps. *Nature Methods* **19**, 1116-1125 (2022).

bioRxiv preprint doi: https://doi.org/10.64898/2026.01.14.699417; this version posted January 16, 2026. The copyright holder for this preprint (which was not certified by peer review) is the author/funder, who has granted bioRxiv a license to display the preprint in perpetuity. It is made available under aCC-BY 4.0 International license.

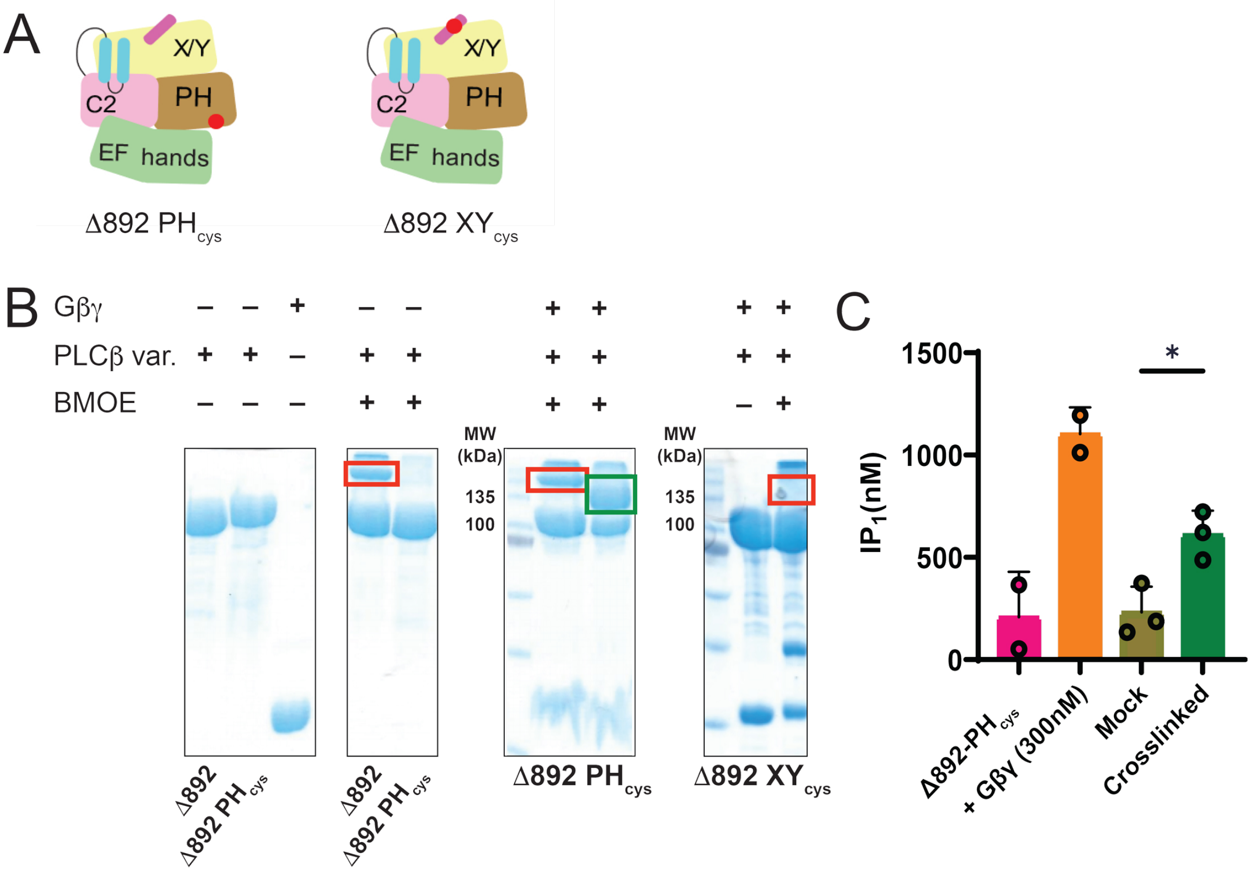

Figure S1. Isolation of a Crosslinked and Functional Gβγ–PLCβ3 complex. (**A**) Schematic of PLCβ3 Δ892 variants used for crosslinking studies. (**B**) Representative crosslinking experiments with Gβγ C68S and PLCβ3 Δ892 variants analyzed by SDS-PAGE. PLCβ3 Δ892 undergoes extensive self-crosslinking (red box, left). Mutation of solvent-exposed cysteines and installation of a single cysteine in the PH domain (E60C) eliminates self-crosslinking and allows crosslinking between Gβγ C68S and PLCβ3 $Δ892_{PH}$ (green box). PLCβ3 $Δ892_{XY}$, which lacks solvent-exposed cysteines with the exception of C516 in the flexible X–Y linker eliminated self-crosslinking but failed to crosslink to Gβγ-C68S. (**C**) BMOE-crosslinked complexes between wild-type Gβγ and PLCβ3 $Δ892_{PH}$ have higher activity in a liposome-based assay than the uncrosslinked control. Data shown are mean of three separate experiments ± SD. Mock crosslinked and crosslinked samples were compared using an unpaired T-test. *, $p < 0.05$.

bioRxiv preprint doi: https://doi.org/10.64898/2026.01.14.699417; this version posted January 16, 2026. The copyright holder for this preprint (which was not certified by peer review) is the author/funder, who has granted bioRxiv a license to display the preprint in perpetuity. It is made available under aCC-BY 4.0 International license.

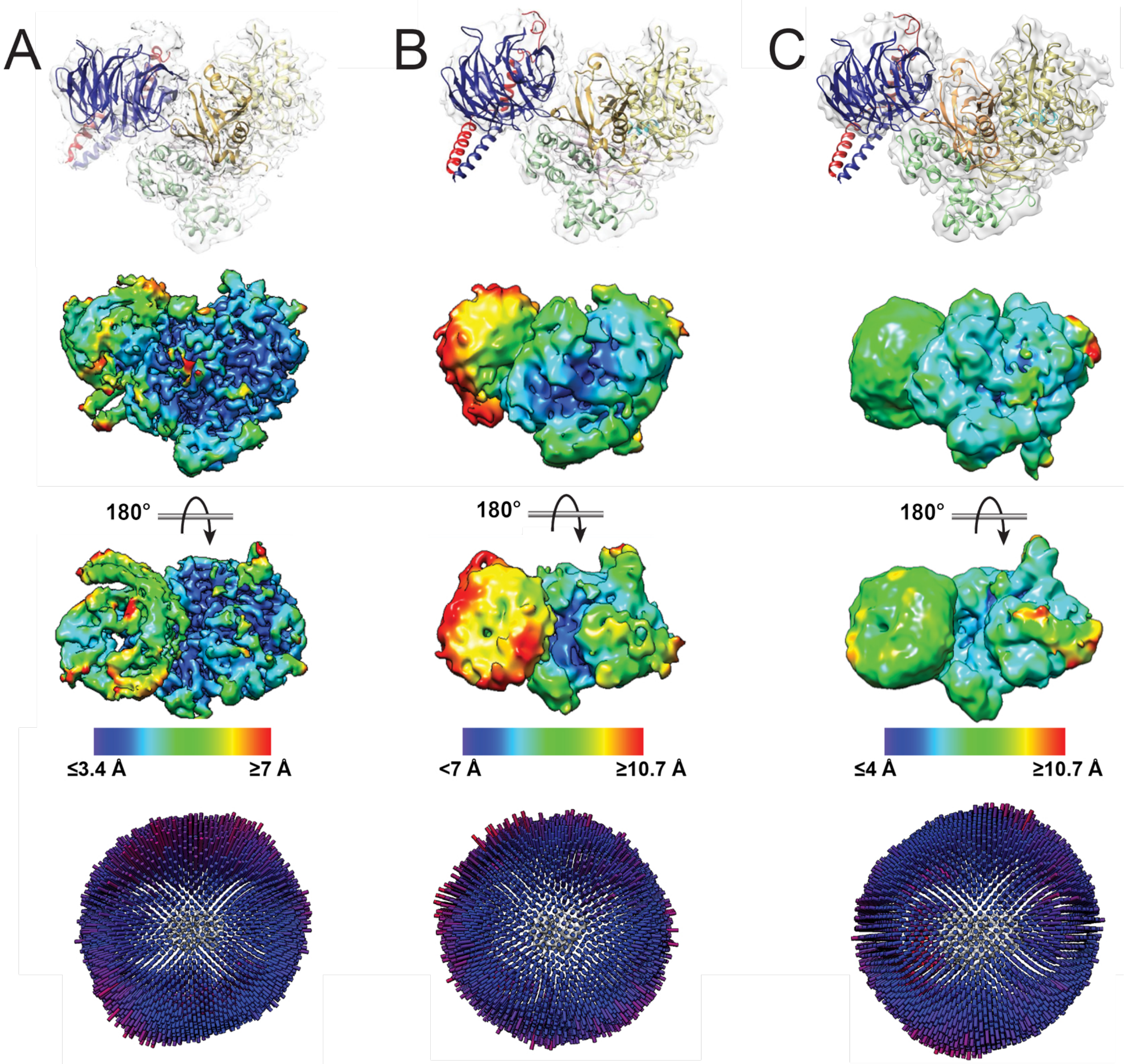

Figure S2. Cryo-EM densities of Gβγ–PLCβ3 Δ892-PH$_{cys}$ complexes. (**A**) *Top.* Model of the larger particle population in the BMOE-crosslinked Gβγ–PLCβ3 Δ892-PH$_{cys}$ reconstruction fit into the cryo-EM density map. *Bottom*. Cryo-EM map colored by local resolution. (**B**) Model of the smaller particle population in the BMOE-crosslinked Gβγ–PLCβ3 Δ892-PH$_{cys}$ reconstruction fit into the cryo-EM density map. ***Bottom.*** Cryo-EM map colored by local resolution. (**C**) *Top.* Model of the BM(PEG)2-crosslinked Gβγ–PLCβ3 Δ892-PH$_{cys}$ reconstruction fit into the cryo-EM density map. *Bottom*. Cryo-EM map colored by local resolution. In all reconstructions, resolution is lower for Gβγ, consistent with a dynamic interface in solution.

bioRxiv preprint doi: https://doi.org/10.64898/2026.01.14.699417; this version posted January 16, 2026. The copyright holder for this preprint (which was not certified by peer review) is the author/funder, who has granted bioRxiv a license to display the preprint in perpetuity. It is made available under aCC-BY 4.0 International license.

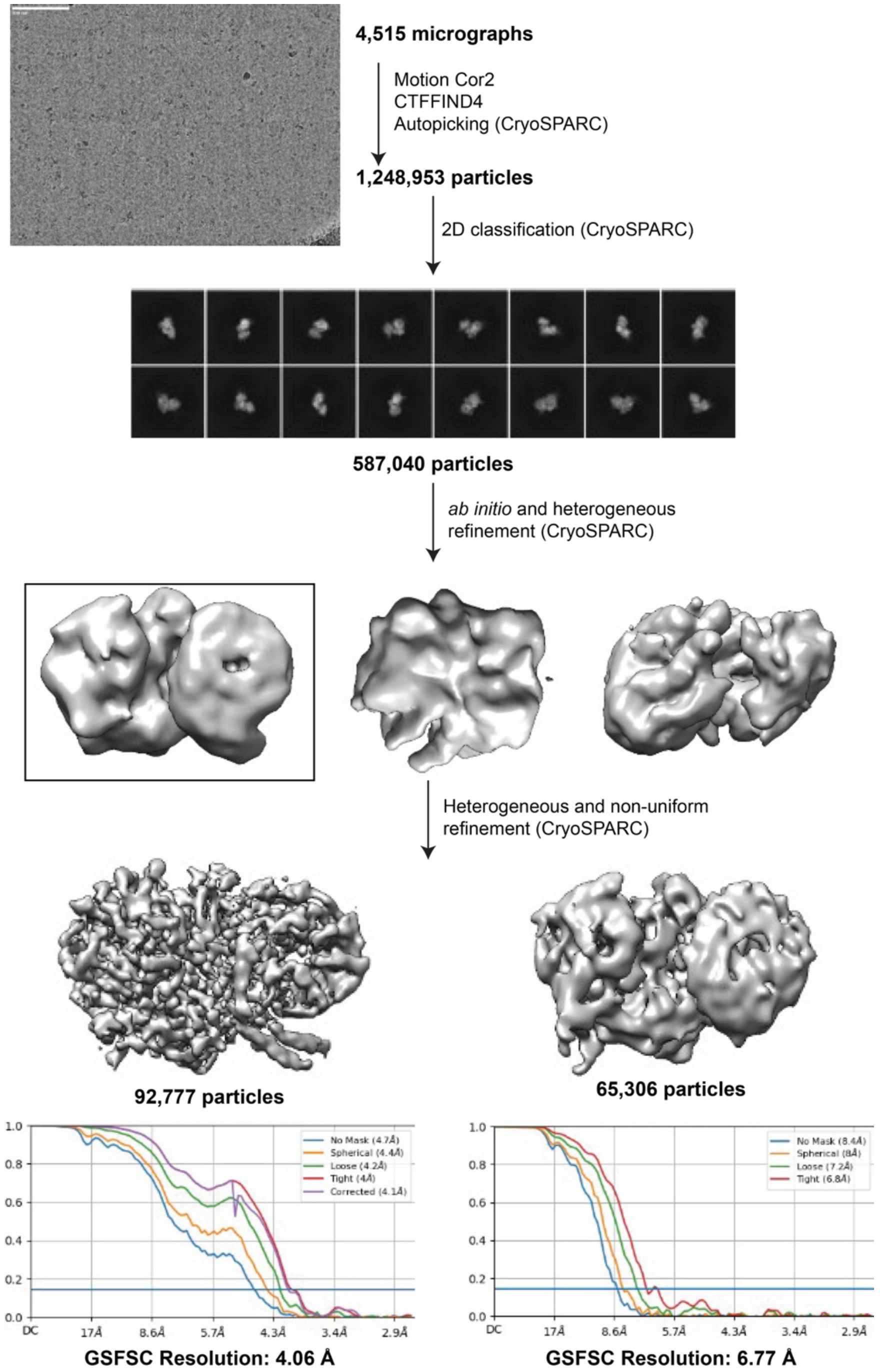

Figure S3. Cryo-EM data workflow and resolution analysis of the BMOE-crosslinked Gβγ–PLCβ3 Δ892-PH$_{cys}$ complex. The workflow, including a representative micrograph, 2D class averages (box size: 276 Å) and Fourier shell correlation (FSC) curves calculated from two independent reconstructions by CryoSPARC(53). The nominal resolution of the resulting map, as defined by the 0.143 cutoff, is indicated by the horizontal blue line.

bioRxiv preprint doi: https://doi.org/10.64898/2026.01.14.699417; this version posted January 16, 2026. The copyright holder for this preprint (which was not certified by peer review) is the author/funder, who has granted bioRxiv a license to display the preprint in perpetuity. It is made available under aCC-BY 4.0 International license.

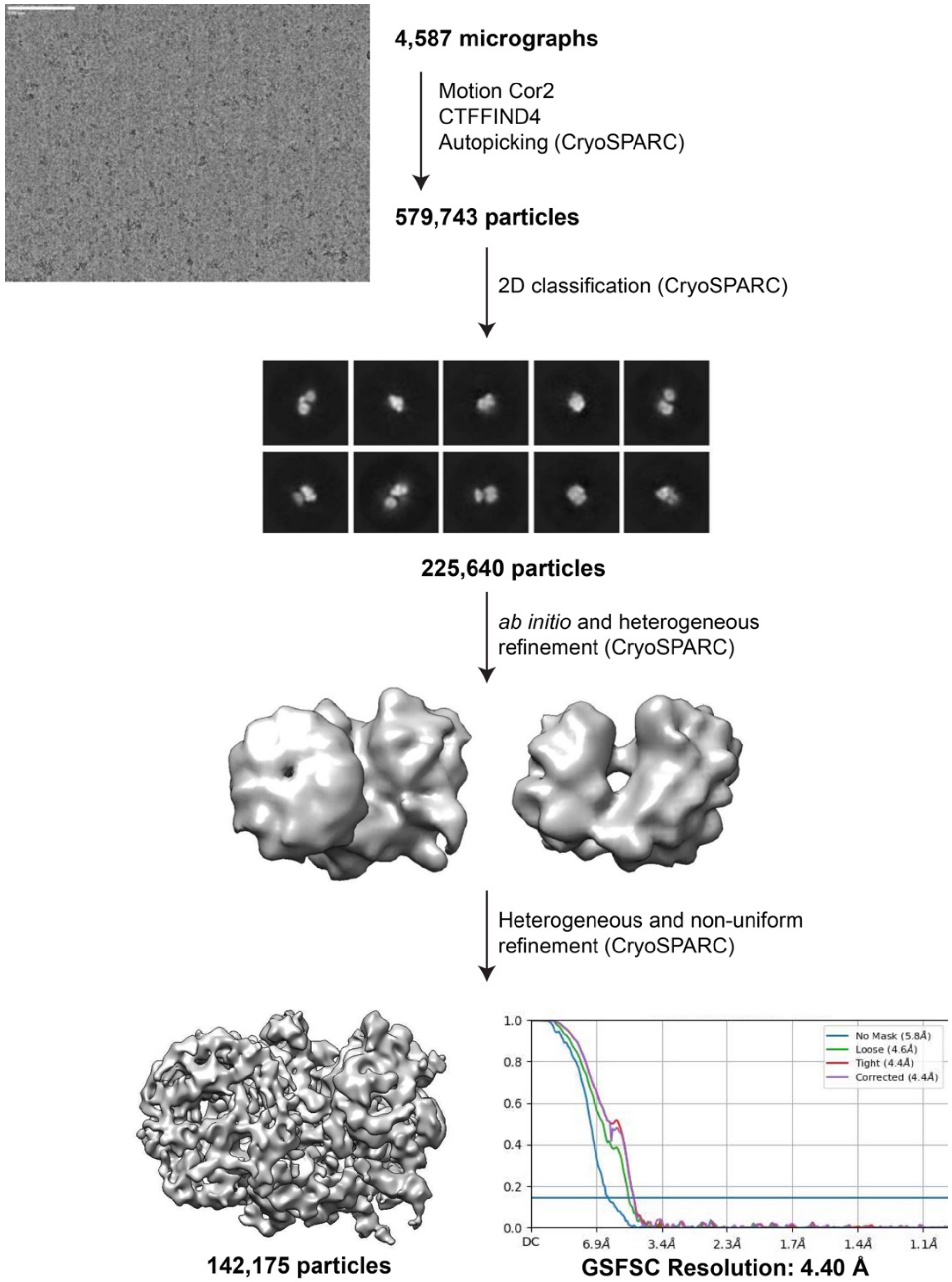

Figure S4. Cryo-EM data workflow and resolution analysis of the BMPEG-crosslinked Gβγ–PLCβ3 Δ892-PH$_{cys}$ complex. The workflow, including a representative micrograph, 2D class averages (box size: 276 Å) and Fourier shell correlation (FSC) curves calculated from two independent reconstructions by CryoSPARC(53). The nominal resolution of the resulting map, as defined by the 0.143 cutoff, is indicated by the horizontal blue line.

bioRxiv preprint doi: https://doi.org/10.64898/2026.01.14.699417; this version posted January 16, 2026. The copyright holder for this preprint (which was not certified by peer review) is the author/funder, who has granted bioRxiv a license to display the preprint in perpetuity. It is made available under aCC-BY 4.0 International license.

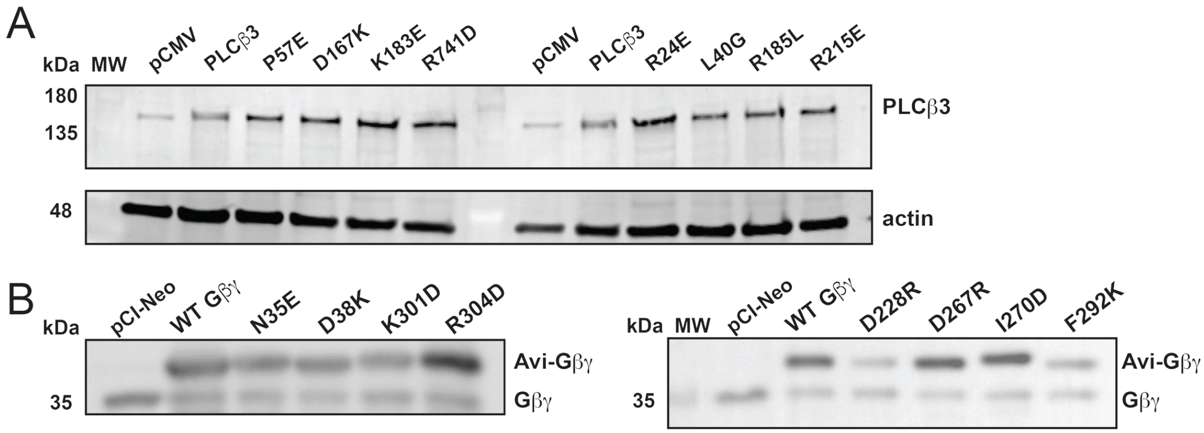

Figure S5. Expression of PLCβ3 and Gβγ mutants. Representative western blot image of cell lysates containing mutants of interest. (**A**) Western blot image of PLCβ3 point mutants. (**B**) Western blot image of Gβ1 mutants. Transfected Gβγ is Avi-tagged (avi-Gβγ) and endogenous Gβ is detected in the untransfected controls.

bioRxiv preprint doi: https://doi.org/10.64898/2026.01.14.699417; this version posted January 16, 2026. The copyright holder for this preprint (which was not certified by peer review) is the author/funder, who has granted bioRxiv a license to display the preprint in perpetuity. It is made available under aCC-BY 4.0 International license.

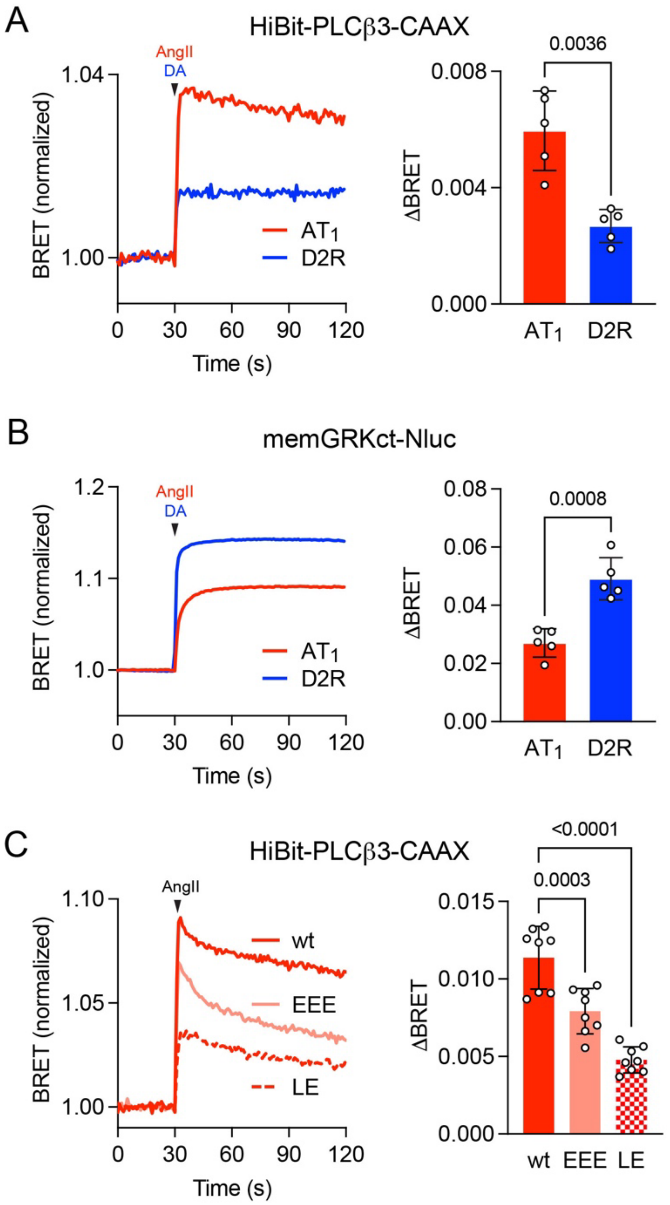

Figure S6. Gα_q binding promotes Gβγ binding to PLCβ3. (**A**) BRET between membrane-anchored HiBit-PLCβ3-CAAX and Venus-Gβγ increases after activation of AT_1 with angiotensin II (AngII; 1 μM) or dopamine D2R receptors with dopamine (DA; 100 μM). Traces represent the average of twenty replicates from five independent experiments. Signals were significantly smaller (p=0.0036) after activation of D2R; Welch's t-test. (**B**) BRET between the Gβγ sensor memGRKct-Nluc and Venus-Gβγ increases after activation of AT_1 or D2R. Traces represent the average of twenty replicates from five independent experiments. Signals were significantly larger (p=0.0008) after activation of D2R; Welch's t-test. Transfection was identical for panels A and B with the substitution of memGRKct-Nluc for HiBit-PLCβ3-CAAX in panel B; Gα_q and Gα_{i1} were overexpressed together with Venus-Gβγ in both panels. (**C**) BRET between HiBit-PLCβ3-CAAX wild-type (wt) and mutants with defective Gα_q binding to the distal CTD (EEE) or proximal CTD (LE). Traces represent the average of 32 replicates from eight independent experiments.

bioRxiv preprint doi: https://doi.org/10.64898/2026.01.14.699417; this version posted January 16, 2026. The copyright holder for this preprint (which was not certified by peer review) is the author/funder, who has granted bioRxiv a license to display the preprint in perpetuity. It is made available under aCC-BY 4.0 International license.

Signals were significantly smaller for both mutants; one-way ANOVA with Dunnett's post-hoc comparisons.

bioRxiv preprint doi: https://doi.org/10.64898/2026.01.14.699417; this version posted January 16, 2026. The copyright holder for this preprint (which was not certified by peer review) is the author/funder, who has granted bioRxiv a license to display the preprint in perpetuity. It is made available under aCC-BY 4.0 International license.

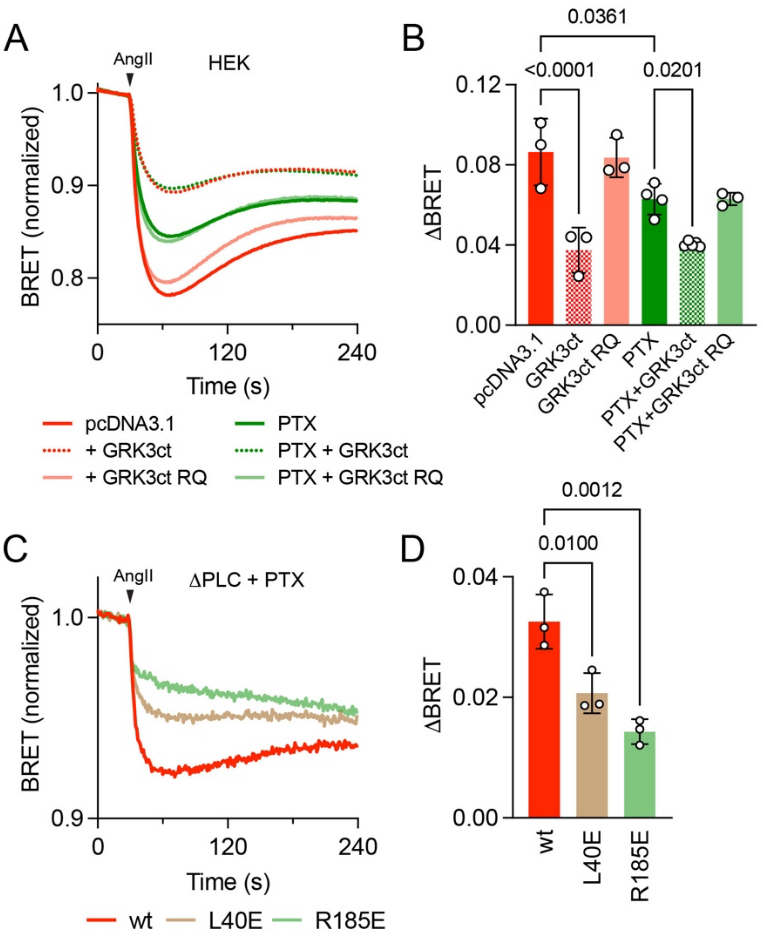

Figure S7. PLCβ-mediated PIP2 hydrolysis is facilitated by Gβγ derived from both G_q and $G_{i/o}$ heterotrimers. (**A**) In HEK cells bystander BRET between Nluc-PH and mem-Venus decreases in response to AT1 activation with AngII (1 μM). Responses are inhibited by GRK3ct, which sequesters Gβγ, but not the binding-defective R587Q mutant (GRKct RQ), both before and after inactivation of $G_{i/o}$ heterotrimers with pertussis toxin (PTX). Traces are the average 12-16 replicates from 3-4 independent experiments. (**B**) Grouped data from the same experiments as panel A; indicated p values are from one-way ANOVA with Tukey's multiple comparisons test. (**C**) In ΔPLC cells expressing PTX, HiBit-PLCβ3 L40E and R185E mutants fail to fully reconstitute AngII-induced PIP2 hydrolysis compared to the wild-type (wt) enzyme; traces are the average of twelve replicates from three independent experiments. (**D**) Grouped data from the same experiments as panel C; indicated p values are from one-way ANOVA with Dunnett's multiple comparisons test.

bioRxiv preprint doi: https://doi.org/10.64898/2026.01.14.699417; this version posted January 16, 2026. The copyright holder for this preprint (which was not certified by peer review) is the author/funder, who has granted bioRxiv a license to display the preprint in perpetuity. It is made available under aCC-BY 4.0 International license.

Table S1. Expression of HiBit-PLCβ3 variants as indicated by total LgBit-complemented luminescence in intact cells.

construct	*raw luminescence (photons)*			*n*
	mean	S.D.	*P* value†	
HiBit-PLCβ3	1.22E+07	6.27E+06	-	10
R24A	1.61E+07	6.51E+06	0.9768	3
R24E	3.25E+06	6.33E+05	0.1537	3
L40E	3.59E+06	1.26E+06	0.1905	3
L40K	2.62E+06	5.09E+05	0.1017	3
D167A	1.56E+07	4.73E+06	0.9928	3
D167G	1.27E+07	3.30E+06	>0.9999	3
R204A	1.85E+07	4.91E+06	0.6269	3
R204E	3.27E+06	5.89E+05	0.156	3
R24E+R204E	3.51E+06	1.30E+06	0.1809	3
R185E	1.23E+07	8.58E+06	>0.9999	4
R215A	1.19E+07	7.40E+06	>0.9999	3
R215E	1.21E+07	4.90E+06	>0.9999	3
L222K	1.37E+07	5.25E+06	>0.9999	4
F285E	8.39E+06	4.37E+06	0.9484	4
K183E	1.67E+07	6.30E+06	0.8662	4

†One-way ANOVA, Dunnett's multiple comparisons test vs. wild-type.

Table S2. Expression of HiBit-PLCβ3-CAAX variants as indicated by total LgBit-complemented luminescence in intact cells.

construct	*raw luminescence (photons)*			*n*
	mean	S.D.	*P* value†	
HiBit-PLCβ3-CAAX	1.54E+08	1.67E+08	-	7
R24A	2.51E+07	1.32E+07	0.1474	4
R24E	3.49E+07	2.05E+07	0.2216	4
L40E	3.65E+07	2.28E+07	0.2356	4
L40K	2.61E+07	1.37E+07	0.1539	4
D167A	2.75E+07	1.48E+07	0.1632	4
D167G	3.03E+07	1.94E+07	0.1833	4
R204A	3.76E+07	2.40E+07	0.2456	4
R204E	4.46E+07	2.76E+07	0.319	4
R24E+R204E	2.98E+07	1.54E+07	0.1797	4
R185E	2.43E+08	1.59E+08	0.719	3
R215A	3.15E+07	2.04E+07	0.1925	4
R215E	3.48E+07	2.49E+07	0.2203	4
L222K	1.91E+08	1.29E+08	0.9998	3
F285E	2.00E+08	1.11E+08	0.9976	3
K183E	1.55E+08	8.66E+07	>0.9999	3

†One-way ANOVA, Dunnett's multiple comparisons test vs. wild-type-CAAX.

bioRxiv preprint doi: https://doi.org/10.64898/2026.01.14.699417; this version posted January 16, 2026. The copyright holder for this preprint (which was not certified by peer review) is the author/funder, who has granted bioRxiv a license to display the preprint in perpetuity. It is made available under aCC-BY 4.0 International license.

Table S3. Angiotensin II-induced BRET between HiBit-PLCβ3-CAAX variants and Venus-Gβγ.

construct	*ΔBRET*			*n*
	mean	S.D.	*P* value†	
HiBit-PLCβ3-CAAX	0.01343	0.003746	-	10
+GRK3ct	-0.000968	0.001502	<0.0001	8
R24A	0.008984	0.001371	0.3104	5
R24E	0.004995	0.00116	0.0002	7
L40E	0.001863	0.00054	<0.0001	5
L40K	0.002092	0.00071	<0.0001	5
D167A	0.0306	0.01035	<0.0001	5
D167G	0.01943	0.004322	0.0517	5
R204A	0.007136	0.001778	0.0350	5
R204E	0.003198	0.000906	<0.0001	7
R24E+R204E	0.002536	0.001165	<0.0001	4
R185E	0.000937	0.000516	<0.0001	3
R215A	0.02478	0.00525	<0.0001	5
R215E	0.02706	0.006269	<0.0001	5
L222K	0.006882	0.00098	0.1052	3
F285E	0.002372	0.000248	0.0003	3
K183E	0.0116	0.002322	0.9984	4

†One-way ANOVA, Dunnett's multiple comparisons test vs. wild-type-CAAX.

Table S4. Angiotensin II-induced PIP2 hydrolysis mediated by HiBit-PLCβ3 variants.

construct	*ΔBRET*			*n*
	mean	S.D.	*P* value†	
HiBit-PLCβ3	0.05223	0.005327	-	11
+GRK3ct	0.02145	0.003139	<0.0001	11
R24A	0.053	0.008833	>0.9999	3
R24E	0.04107	0.004471	0.0030	5
L40E	0.02582	0.004777	<0.0001	5
L40K	0.02727	0.003389	<0.0001	5
D167A	0.07282	0.001987	<0.0001	3
D167G	0.06207	0.01019	0.0707	3
R204A	0.04634	0.002524	0.6770	3
R204E	0.03782	0.007242	<0.0001	5
R24E+R204E	0.03578	0.0041	<0.0001	4
R185E	0.01643	0.001868	<0.0001	3
R215A	0.06183	0.005277	0.0843	3
R215E	0.06154	0.006815	0.1041	3
L222K	0.02474	0.001306	<0.0001	3
F285E	0.01552	0.00182	<0.0001	3
K183E	0.03727	0.009687	0.0007	3

†One-way ANOVA, Dunnett's multiple comparisons test vs. wild-type.

bioRxiv preprint doi: https://doi.org/10.64898/2026.01.14.699417; this version posted January 16, 2026. The copyright holder for this preprint (which was not certified by peer review) is the author/funder, who has granted bioRxiv a license to display the preprint in perpetuity. It is made available under aCC-BY 4.0 International license.

Table S5. Angiotensin II-induced BRET between HiBit-PLCβ3 variants and Gαq-Venus.

construct	*ΔBRET*			*n*
	mean	S.D.	*P* value†	
HiBit-PLCβ3	0.07705	0.01942	-	3
R24A	0.08169	0.01774	>0.9999	3
R24E	0.09379	0.01895	0.8153	3
L40E	0.105	0.01828	0.2442	3
L40K	0.07575	0.008115	>0.9999	3
D167A	0.1023	0.01475	0.3513	3
D167G	0.05385	0.01087	0.4539	3
R204A	0.0951	0.02177	0.7455	3
R204E	0.1066	0.01497	0.1925	3
R24E+R204E	0.1008	0.01792	0.4274	3
R185E	0.1094	0.02068	0.1247	3
R215A	0.1116	0.01287	0.0854	3
R215E	0.08856	0.01038	0.9817	3
L222K	0.08054	0.01358	>0.9999	3
F285E	0.05337	0.001885	0.4289	3
K183E	0.1021	0.007104	0.3642	3

†One-way ANOVA, Dunnett’s multiple comparisons test vs. wild-type.

Table S6. Angiotensin II-induced BRET between HiBit-PLCβ3 variants and mem-Venus.

construct	*ΔBRET*			*n*
	mean	S.D.	*P* value†	
HiBit-PLCβ3	0.03366	0.003477	-	3
R24A	0.03012	0.00526	0.9390	3
R24E	0.03258	0.002824	>0.9999	3
L40E	0.03697	0.003716	0.9607	3
L40K	0.03456	0.002387	>0.9999	3
D167A	0.03445	0.003044	>0.9999	3
D167G	0.02822	0.003124	0.5771	3
R204A	0.03021	0.006573	0.9481	3
R204E	0.03401	0.004711	>0.9999	3
R24E+R204E	0.03131	0.003755	0.9978	3
R185E	0.02671	0.002865	0.2875	3
R215A	0.0381	0.004039	0.7947	3
R215E	0.03365	0.005771	>0.9999	3
L222K	0.03058	0.003968	0.9770	3
F285E	0.02902	0.002389	0.7545	3
K183E	0.02648	0.002579	0.2535	3

†One-way ANOVA, Dunnett’s multiple comparisons test vs. wild-type.
